# Environmental influences on the accumulation of medicinal active components and metabolites of *Plantago asiatica L.* seeds from different cultivation sites

**DOI:** 10.3389/fpls.2026.1775798

**Published:** 2026-02-24

**Authors:** Xinlin Peng, Qian Qin, Yan Luo, Kailin Qiao, Yu Zhu, Bo Wang, Zhou Zhang, Luxin Xie, Shouwen Zhang

**Affiliations:** 1 Pharmacy School, Jiangxi University of Chinese Medicine, Nanchang, China; 2 Research Center for Traditional Chinese Medicine Resources and Ethnic Minority Medicine, Jiangxi University of Chinese Medicine, Nanchang, China; 3 Jiangxi Key Laboratory for Sustainable Utilization of Chinese Materia Medica Resources, Jiangxi University of Chinese Medicine, Nanchang, China

**Keywords:** environmental factors, lipids, medicinal plants, metabolome, *Plantago asiatica L.* seeds, terpenoids

## Abstract

**Introduction:**

Soil nutrients and climate critically regulate the accumulation of bioactive components in *Plantago asiatica L.* seeds, but their regulatory mechanisms are still unclear. Thus, this study aims to explore the regulatory roles of these factors in *Plantago asiatica L.* seeds.

**Method:**

A combination of multiple techniques including High Performance Liquid Chromatography (HPLC), Gas Chromatography-Mass Spectrometry (GC-MS), and Liquid Chromatography-Mass Spectrometry (LC-MS) was employed to study the regulatory effects of soil nutrients and climate on the growth of *Plantago asiatica L.* seeds from Jiangxi and Sichuan provinces.

**Result:**

Notably, the key anti-inflammatory components, geniposidic acid and acteoside, were significantly more abundant in seeds from Jiangxi, which supports the recognition that Jiangxi-sourced *Plantago asiatica L.* seeds have “superior medicinal quality”. Specifically, the high temperature, abundant precipitation, and soil rich in available phosphorus (P), iron (Fe), and manganese (Mn) in Jiangxi may upregulate lipid metabolism through the linoleic acid pathway. In contrast, the mild climate, significant seasonal variation in precipitation, and soil characterized by high available boron (B), exchangeable magnesium (Mg), and slight alkalinity in Sichuan may upregulate terpenoids metabolism through the retinoid metabolism and transport pathway, as well as the accumulation of specific volatiles including (-)-camphene and (Z)-carveol.

**Conclusion:**

Collectively, these soil and climate factors synergistically shape the differences in metabolomic profiles and medicinal quality of *Plantago asiatica L.* seeds. This study provides a theoretical basis for screening high-quality production areas and formulating standardized cultivation strategies.

## 1 Introduction

*Plantago asiatica L.*, a member of the Plantaginaceae family, is a medicinal herb with a long history of utilization in the traditional medicine systems of East Asia (Huan et al., 2024). Its dried mature seeds, which exhibit pharmacological effects including clearing heat, promoting diuresis, resolving dampness, stopping diarrhea, improving eyesight, and eliminating phlegm (Tong et al., 2019; Wen et al., 2023), are not only widely used as an adjuvant therapeutic agent in clinical practice for urinary system diseases, digestive disorders (Caporali et al., 2022; Ji-Ping et al., 2021), and ocular discomfort but also demonstrate tremendous development potential in the field of functional health products (Liu et al., 2024; Tamura et al., 2022).

*Plantago asiatica L.* seeds are predominantly cultivated in China, with a national planting distribution characterized by concentration in core producing areas. Jiangxi Province, a traditional and genuine producing region, contributes over 70% to the national total output, with its core planting areas concentrated in Ji’an, Zhangshu, and other regions. The medicinal seeds produced here are highly sought after by Chinese materia medica slice manufacturers due to their superior quality. Sichuan Province ranks as the second-largest producing area, with its cultivation primarily distributed in Shifang, Zigong, and other areas. Traditionally, the cultivation methods of *P. asiatica* are largely similar across regions: it is generally grown in sandy loam, with direct seeding for seedling raising in September, transplanting to fields in November; base fertilizer mainly consists of organic fertilizer and compound fertilizer, followed by two topdressings with compound fertilizer in March to April of the following year, and harvesting in May. The whole process relies on local natural conditions, with irrigation adjusted according to seasonal rainfall, and no complex artificial management measures—resulting in minimal inter-regional differences in cultivation practices. Nevertheless, the quality formation mechanisms of indigenous *P. asiatica* seeds from Jiangxi remain elusive (Wu et al., 2023). Although it is well-documented that local environmental factors—such as soil organic matter content, temperature, and humidity—are associated with the quality of these seeds (Daneshmandi et al., 2025; Guo et al., 2013), the synergistic regulatory effects of such factors on the biosynthetic pathways of bioactive components in *P. asiatica* seeds have not been elucidated.

The quality variation of genuine medicinal materials is essentially attributed to the synergistic effects of genetic and environmental factors (Liu et al., 2020; Wang et al., 2025), among which climatic conditions and soil physicochemical properties serve as the core environmental drivers that regulate the accumulation of secondary metabolites in medicinal plants (Huang et al., 2025; Li et al., 2022; Yang et al., 2024). Given that the cultivation methods of *P. asiatica* are largely similar across regions, soil and climate factors are regarded as the primary drivers of inter-regional quality variations. In recent decades, metabolomics has emerged as a powerful tool for elucidating the quality formation mechanisms of traditional Chinese medicinal materials (TCMM) (Guo et al., 2022; Zhang et al., 2023, 2025). The integrated application of technologies, including high-performance liquid chromatography (HPLC) (Bārzdiņa et al., 2022), gas chromatography-mass spectrometry (GC-MS), and liquid chromatography-mass spectrometry (LC-MS), enables comprehensive profiling of bioactive components, volatile metabolites (Yang et al., 2023), and non-volatile metabolites (Fang et al., 2023). Meanwhile, the interdisciplinary integration of environmental science (Jangpangi et al., 2025) and metabolomics has become a cutting-edge research direction in the field of medicinal plants. Climatic and soil physicochemical factors directly influence the accumulation of secondary metabolites by regulating plant enzyme activities and metabolic flux (Qaderi et al., 2023a)—a mechanism that has been validated in studies on medicinal plants such as rhubarb (Zhang et al., 2025) and wolfberry (Sun et al., 2025).

While prevailing research across medicinal plant taxa has suffered from fragmented perspectives, most studies either isolate the impacts of single environmental factors or conduct disconnected metabolomic profiling, failing to establish systematic, mechanistic links between combined climatic and soil cues, metabolic flux dynamics and secondary metabolite biosynthesis (Jangpangi et al., 2025; Qaderi et al., 2023a). This research gap is further exemplified by existing studies on *P. asiatica* seeds, which are largely confined to individual chemical component identification (Niu et al., 2017), pharmacological effect verification (Liu et al., 2025) or preliminary exploration of single environmental factors (Ishikawa et al., 2007).

To address these knowledge gaps, the present study targets the two major *P. asiatica* seeds producing areas (Jiangxi and Sichuan Provinces). We integrated three advanced technologies—high-performance liquid chromatography (HPLC), gas chromatography-mass spectrometry (GC-MS), and liquid chromatography-mass spectrometry (LC-MS)—with soil physicochemical property determination methods. Specifically, this integration aimed to achieve precise detection and identification of core bioactive components, volatile organic compounds (VOCs), and non-volatile metabolites in *P. asiatica* seeds. By incorporating climatic and ecological factors as well as soil physicochemical properties, we constructed a correlation network between the climate-soil dual drivers and both the metabolomic profiles and bioactive component contents of *P. asiatica* seeds. Through this approach, we identified the core driving factors underlying metabolomic differentiation and the key metabolic pathways involved, thereby systematically elucidating the impact of the ecological environment on the quality of genuine *P. asiatica* seeds. This study validates and provides a scientific basis for the traditional perception that Jiangxi-sourced *P. asiatica* seeds possess superior medicinal quality. Furthermore, it offers multi-dimensional technical support and theoretical references for screening high-quality *P. asiatica* seeds producing areas, regulating cultivation environments, and enhancing medicinal quality. Importantly, this research holds significant exemplary value for advancing systematic studies on the quality formation mechanisms of genuine traditional Chinese medicinal materials (TCMM).

## 2 Materials and methods

### 2.1 Sample and chemicals

#### 2.1.1 Sample collection and preparation

**Figure 1:**
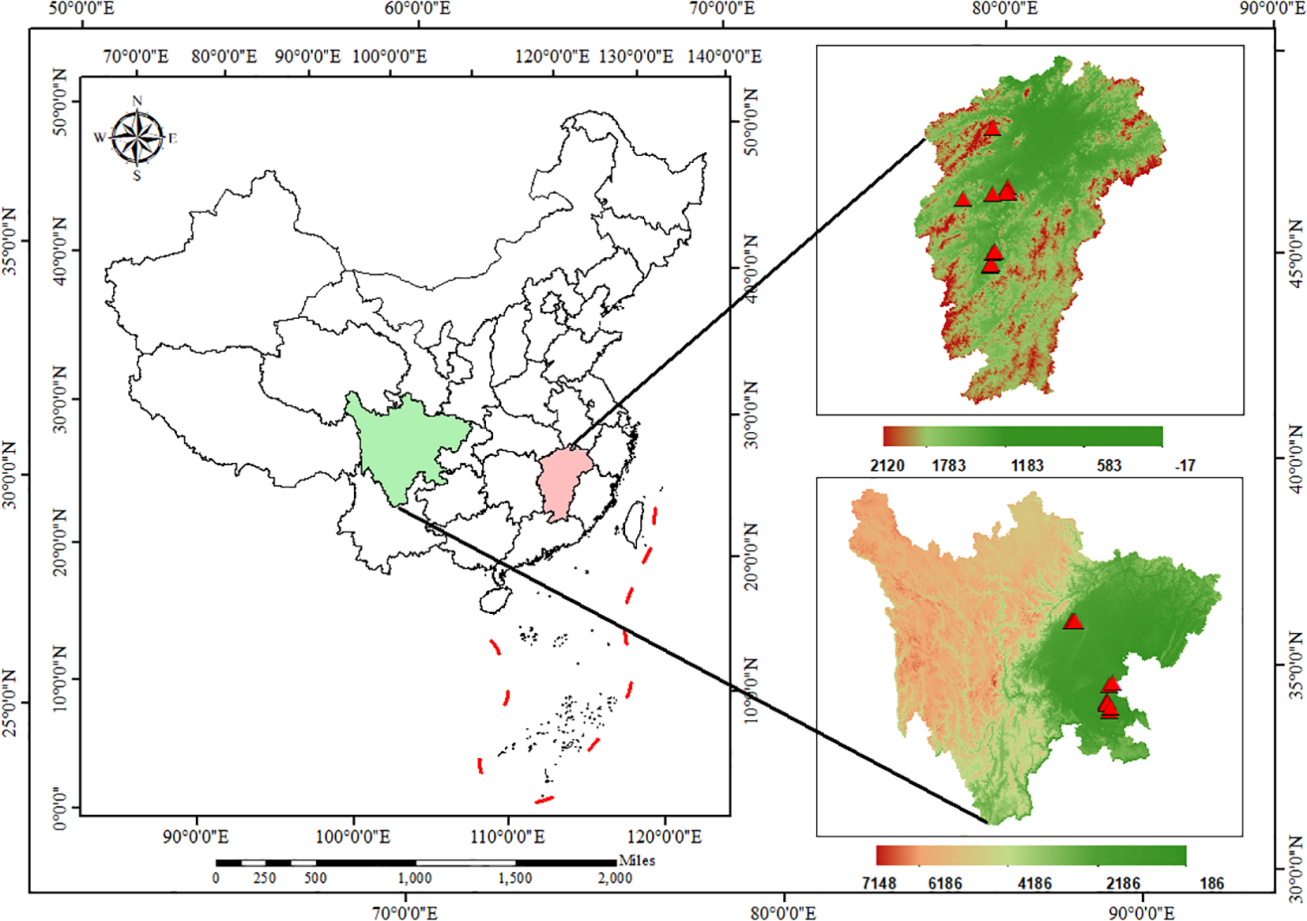
The geographical distribution of 26 *Plantago asiatica L.* seeds cultivation areas in Jiangxi Province and Sichuan Province, China: the “pink” area represents Jiangxi Province, the “green” area represents Sichuan Province, and the red triangles denote sampling sites.

A total of 26 *Plantago asiatica L.* seed samples were collected during May 12–20, 2025 (Blaise et al., 2016; Gori et al., 2020), covering the core production areas of Jiangxi Province (14 samples, including Ji’an, Zhangshu, Xinyu, and other regions) and Sichuan Province (12 samples, including Shifang, Zigong, Luzhou, and other regions), with an interval of more than 20 square kilometers between each sampling site. Each sample was prepared with three biological replicates, and all samples were collected during the maturation stage of *P. asiatica* seeds (**Figure 1**, **Supplementary Table S1**).

All seed samples were taxonomically identified by Professor Zhang Shouwen from Jiangxi University of Chinese Medicine as the dried mature seeds of *Plantago asiatica L.* The collected *P. asiatica* seeds were processed as follows: first sun-dried to constant weight, then air-screened to remove impurities, and subsequently ground into coarse powder. The coarse powder was further sieved through a No. 3 Pharmacopoeia sieve to obtain homogeneous fine powder, which was stored in a desiccator at room temperature for subsequent experimental analysis.

#### 2.1.2 Rhizosphere soil sample collection and preparation

Correspondingly, rhizosphere soil samples (0–20 cm soil layer) were collected in a one-to-one correspondence with the *P. asiatica* seed samples. At each sampling site, the five-point sampling method (J. Yang et al., 2024) was employed to ensure the representativeness of soil samples: five sub-samples (each about 400 g) were collected around the rhizosphere of target plants, with a total weight of approximately 2 kg, and thoroughly mixed to form one composite soil sample. After collection, stones, plant residues, and other impurities were manually removed from the composite soil samples. The processed soil samples were naturally air-dried in a well-ventilated and shaded environment to avoid direct sunlight (to prevent the volatilization of volatile components and changes in physicochemical properties). After drying, the soil samples were crushed and passed through a 20-mesh sieve to achieve particle uniformity. Each sieved soil sample was divided into two portions: one portion was immediately used for the determination of soil physicochemical indices, and the other portion was sealed in a polyethylene bag and stored at room temperature for backup analysis (Li et al., 2022).

#### 2.1.3 Chemical reagents

The standard substances used in this study were as follows: geniposidic acid (batch number: JOT-10449) was purchased from Chengdu Pufeide Biotechnology Co., Ltd. (Chengdu, Sichuan, China); acteoside (batch number: JOT-10007) was also obtained from Chengdu Pufeide Biotechnology Co., Ltd.; isacteoside (batch number: RFS-Y07302312022) and luteoside (batch number: RFS-M02502312027) were purchased from Chengdu Ruifensidan Biotechnology Co., Ltd. (Chengdu, Sichuan, China); ferulic acid (batch number: B20007-20mg) was supplied by Shanghai Yuanye Biotechnology Co., Ltd. (Shanghai, China). The purity of all above-mentioned standard substances was ≥ 98%.Chromatographic grade methanol was purchased from Merck Biotechnology (China) Co., Ltd. (Shanghai, China). Analytical grade methanol and acetic acid were obtained from Xilong Science Co., Ltd. (Guangzhou, Guangdong, China). Ultrapure water used in all experiments was prepared using an ultrapure water purification system (Millipore Corporation, Bedford, MA, USA).

### 2.2 Acquisition of climatic factors

Nineteen climatic factors were used to characterize the ecological conditions of *P. asiatica* seeds cultivation regions, and their data were sourced from two authoritative databases: 1. WorldClim Global Climate Database (http://worldclim.org/version2; accessed on September 7th, 2025): Gridded data spatial resolution is 30 seconds (about 1 km²). Data preprocessing adopted the high-resolution climate layer construction methodology (Poggio et al., 2018) with reference to the foundational framework (Fick and Hijmans, 2017). Temperature-related variables are stored as “°C×10” (scaling factor 0.1 required to restore actual °C values), and precipitation-related variables are in “mm” (no scaling factor needed).2. CliMond Global Bioclimatic Modeling Database (Ramirez-Cabral et al., 2017; https://www.climond.info/; accessed on September 7th, 2025): Interpolated climate surfaces have a spatial resolution of 10′ (about 18 km²), consistent with its global conformal design. All climatic variables are used actual units (temperature: °C, precipitation: mm) without requiring scaling factors.

Based on the longitude, latitude, and altitude of each *P. asiatica* seed sampling sites, the 19 climatic factors corresponding to each production area were extracted using the GMPGIS (Geographic Information System for Global Medicinal Plants) platform. These factors (units consistent with the above databases) included: annual mean temperature (ANNT, °C), mean monthly temperature range (MOND, °C), isothermality (ISOT, dimensionless, ratio of mean diurnal range to annual temperature range), temperature seasonality (TEMP, coefficient of variation, dimensionless), maximum temperature of the warmest month (HTMT, °C), minimum temperature of the coldest month (CLMT, °C), annual temperature range (ANNR, °C), mean temperature of the wettest quarter (WETQ, °C), mean temperature of the driest quarter (DRYQ, °C), mean temperature of the warmest quarter (HTQQ, °C), mean temperature of the coldest quarter (CLQQ, °C), annual precipitation (ANNP, mm), precipitation of the wettest month (WETM, mm), precipitation of the driest month (DRYM, mm), precipitation seasonality (PREC, coefficient of variation, dimensionless), maximum precipitation of the wettest quarter (WETP, mm), precipitation of the driest quarter (DRYP, mm), precipitation of the warmest quarter (HTQP, mm), and precipitation of the coldest quarter (CLQP, mm) (**Supplementary Table S2**).

### 2.3 Determination of soil physicochemical indices

Fifteen key soil physicochemical indices were determined strictly in accordance with Chinese national standard methods, with specific determination methods and corresponding standard numbers as follows: total nitrogen (Kjeldahl method, LY/T 1228-2015); total phosphorus and available phosphorus (molybdenum-antimony colorimetric method, LY/T 1232–2015 and HJ 704-2014, respectively); total potassium and available potassium (flame photometric method, LY/T 1234–2015 and NY/T 889-2004, respectively); soil organic matter (potassium dichromate volumetric method, LY/T 1237-1999); available zinc, available iron, and available manganese (DTPA extraction method, NY/T 890-2004); available boron (curcumin colorimetric method, LY/T 1258-1999); exchangeable calcium and exchangeable magnesium (atomic absorption spectrophotometry, LY/T 1245-1999); soil pH (potentiometric method, HJ 962-2018); and soil electrical conductivity (electrode method, HJ 802-2016)(**Supplementary Table S3**).

### 2.4 HPLC analysis for bioactive components

Accurately 1.0 g of *P. asiatica* seeds powder (passed through a No. 3 Pharmacopoeia sieve) was weighed and transferred into a stoppered conical flask. Then, 20 mL of methanol was precisely added, and the total mass of the flask and its contents was recorded. The mixture was ultrasonically extracted at 250 W and 40 kHz for 1 h. After extraction, the flask was cooled to room temperature, and methanol was supplemented to restore the lost mass. The extracted solution was transferred to a centrifuge tube and centrifuged at 7000 r·min^-^¹ for 10 min. The supernatant was carefully collected and filtered through a 0.45 μm microporous organic filter membrane to obtain the test solution, which was stored at 4 °C in the dark until HPLC analysis.

HPLC analysis was carried out using a Shimadzu LC-20A liquid chromatograph (Shimadzu Corporation, Kyoto, Japan) equipped with a UV-Vis detector. Chromato-graphic separation was achieved on an Agilent ZORBAX SB-C18 column (4.6 mm × 250 mm, 5 μm). The mobile phase was composed of phase A (0.1% acetic acid aqueous solution) and phase B (methanol), with a gradient elution program optimized as follows: 0.01–5.00 min, 5% B (isocratic elution); 5.00–25.00 min, 5%–25% B (linear gradient); 25.00–40.00 min, 25%–40% B (linear gradient); 40.00–45.00 min, 40%–60% B (linear gradient). The flow rate was maintained at 1.0 mL·min^-^¹, the column temperature was set at 30 °C, the injection volume was 1 μL, and the detection wavelength was 254 nm. The calibration curves for target bioactive components were established with standard substances, and all showed good linearity (correlation coefficient r > 0.999), which was sufficient for quantitative analysis.

### 2.5 GC-MS analysis for volatile organic compounds

Accurately 5.0 g of *P. asiatica* seeds powder was weighed and transferred into a 20 mL headspace vial. The vial was immediately sealed with a polytetrafluoroethylene (PTFE)-lined butyl rubber stopper and an aluminum crimp cap to prevent the loss of volatile components, and the sealed sample was ready for headspace GC-MS analysis.

Headspace GC-MS analysis was performed using an Agilent 7890B gas chromato-graph coupled with an Agilent 5977A mass selective detector (Agilent Technologies, Santa Clara, CA, USA). The headspace equilibrium parameters were set as follows: equilibrium time of 0.5 min, and transfer line temperature maintained at 280 °C. The total GC cycle time was 48.5 min. Chromatographic separation was achieved on an Agilent 19091S-433 HP-5MS capillary column (30 m × 250 μm × 0.25 μm, 5% phenylmethylsiloxane stationary phase). The injection port temperature was set at 250 °C, and high-purity helium (purity ≥ 99.999%) was used as the carrier gas with a constant flow rate of 1.2014 mL/min. The split ratio was 10:1. The programmed temperature rise procedure was optimized as follows: initial temperature of 60 °C (held for 0 min); increased to 100 °C at a rate of 2 °C/min (held for 0 min); further increased to 140 °C at a rate of 5 °C/min (held for 0 min); finally increased to 210 °C at a rate of 4 °C/min (held for 3 min). Mass spectrometry conditions were: electron ionization (EI) source with electron energy of 70 eV; ion source temperature of 230 °C; quadrupole temperature of 150 °C; solvent delay time of 3 min; mass scan range of m/z 35–450.

### 2.6 LC-MS analysis for metabolomic profiling

Accurately 1.0 g of *P. asiatica* seeds powder was weighed and transferred into a stoppered conical flask. Then, 10 mL of methanol (chromatographic grade) was precisely added, and the total mass of the flask and its contents was recorded. The mixture was ultrasonically extracted at 250 W and 40 kHz for 1 h. After extraction, the flask was cooled to room temperature, and methanol was supplemented to restore the mass lost during ultrasonication. The extracted solution was transferred to a centrifuge tube and centrifuged at 7000 r·min^-^¹ for 10 min. The supernatant was collected and filtered through a 0.45 μm microporous organic filter membrane, and the resulting filtrate was used as the test solution. All test solutions were stored at 4 °C until LC-MS analysis to avoid metabolite degradation.

LC-MS analysis was performed using a Shimadzu high-performance liquid chromatography (HPLC) system (equipped with LC30AD dual pumps, SIL30AC autosampler, and CTO30A column oven) coupled with an AB Sciex TripleTOF 5600+ mass spectrometer (AB Sciex, Framingham, MA, USA).

Chromatographic conditions were optimized as follows: column temperature maintained at 40 °C; injection volume of 2.0 μL; mobile phase composed of phase A (0.1% formic acid aqueous solution) and phase B (acetonitrile, LC-MS grade), with a gradient elution program based on Pump B: 0.01–5.00 min, 5%–10% B; 5.00–18.00 min, 10%–25% B; 18.00–23.00 min, 25%–65% B; 23.00–35.00 min, 65%–98% B; 35.00–37.00 min, 98% B (isocratic elution); 37.00–37.10 min, 98%–5% B; 37.10–40.00 min, 5% B (equilibration). The flow rate was set at 0.3 mL·min^-^¹ throughout the analysis.

Mass spectrometry conditions were configured with electrospray ionization (ESI) source operating in both positive and negative ion modes: ion source temperature (TEM) = 500.0 °C; spray voltage (ISVF) = 5.5 kV (positive mode) and 4.5 kV (negative mode); declustering potential (DP) = 100.0 eV (positive mode) and -100.0 eV (negative mode); curtain gas (CUR) flow rate = 40 mL·min^-^¹; auxiliary gas 1 (GS1) and auxiliary gas 2 (GS2) flow rates = 50 mL·min^-^¹ each.

### 2.7 Data processing and statistical analysis

Metabolomics data processing: The raw data was subjected to peak extraction, alignment, and redundancy removal using the MS-DIAL software (https://lipidmaps.org/resources/tools/16?task=4.3). Principal Component Analysis (PCA) was performed for preliminary multivariate statistical analysis to reflect the overall separation trend of samples; Redundancy Analysis (RDA) was used to explore the correlation between soil factors and metabolite profiles. The stability of the method was evaluated using Quality Control (QC) samples, with a Relative Standard Deviation (RSD) < 3%. Differential metabolites were screened using the Orthogonal Partial Least Squares-Discriminant Analysis (OPLS-DA) model with criteria of Variable Importance in Projection (VIP) > 1, t-test p-value (P) < 0.05, and Fold Change (FC) ≥ 2 or FC ≤ 0.5 (Huang et al., 2021). The core role of this screening step is to accurately distinguish metabolic characteristic differences among different sample groups, identify key secondary metabolites related to the research objectives (e.g., effects of soil/climatic factors, differences in sample characteristics), and provide core research targets for subsequent metabolic pathway analysis, clarification of physiological mechanisms, and verification of causal relationships. Moreover, all metabolites discussed in this article are secondary metabolites. Metabolic pathway annotation was performed using the Kyoto Encyclopedia of Genes and Genomes (KEGG) database (https://www.genome.jp/kegg/; Kanehisa et al., 2017). Climate and soil factor data were standardized.

Statistical analysis and data visualization were performed using the following software and platforms: “Similarity Evaluation System for Chromatographic Fingerprint of Traditional Chinese Medicine” (2012 Edition, used to calculate the similarity of HPLC samples), IBM SPSS Statistics 27, OriginPro 2024, Maiwei Cloud Platform (https://cloud.metware.cn/#/home), BioRender (https://www.biorender.dev), and Microsoft Excel (2019 Edition), all of which are publicly available.

## 3 Results

### 3.1 HPLC analysis results

High-performance liquid chromatography (HPLC) was employed to detect 26 batches of *P. asiatica* seed samples, leading to the acquisition of a control spectrum containing 23 common peaks (**Supplementary Figure S1A**). Through comparative analysis with reference substances and qualitative identification based on retention time, a total of five common chromatographic peaks were successfully characterized, namely geniposidic acid (10 min), ferulic acid (24.1 min), acteoside (27.2 min), luteoloside (29.5 min), and isoacteoside (31.4 min) (**Supplementary Figure S1B**). Subsequently, the similarity between the fingerprint spectra of the 26 batches of *P. asiatica* seeds samples and the established control spectrum was evaluated. The similarity values ranged from 0.977 to 1.000 (**Supplementary Table S2**), indicating that the overall chemical composition of *P. asiatica* seeds samples from different producing areas was relatively consistent, and the quality of the medicinal material remained stable across batches.

Notably, the contents of characteristic chemical components in *P. asiatica* seeds varied among different producing areas. The content ranges of the identified components were as follows (**Supplementary Table S4**):eniposidic acid (0.614%–1.215%), acteoside (0.698%–1.294%), isoacteoside (0.049%–0.123%), luteoloside (0.036%–0.093%), and ferulic acid (0.029%–0.067%). Among these components, the contents of acteoside and luteoloside in *P. asiatica* seeds from Jiangxi Province were generally higher than those from Sichuan Province, with a statistically notable difference observed for acteoside (p<0.05) (**Supplementary Figure S2**).

### 3.2 GC-MS analysis results

**Figure 2:**
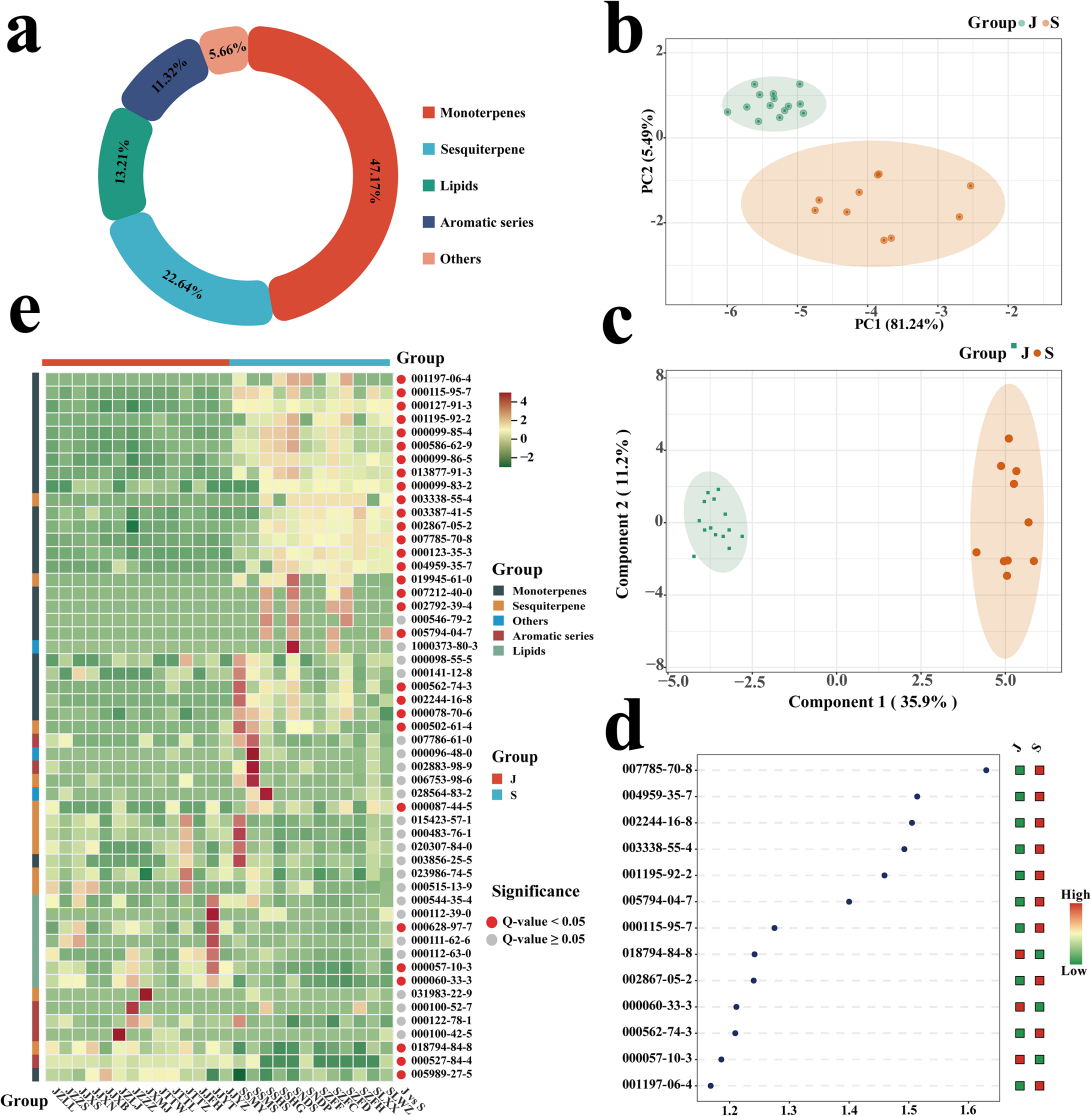
Pie charts of classification **(A)**, principal component analysis (PCA) plots **(B)**, orthogonal projections to latent structures-discriminant analysis (OPLS-DA) plots **(C)**, variable importance in projection (VIP) plots **(D)**, and hierarchical clustering analysis (HCA) heatmaps **(E)** of volatile metabolites detected in *Plantago asiatica L.* seeds from different geographical origins. Group “J” represents Jiangxi Province and Group “S” represents Sichuan Province.

Volatile organic compounds (VOCs) are the primary contributors to the odor characteristics of medicinal materials, and variations in the types and concentrations of VOCs result in distinct odor profiles among *P. asiatica* seeds from different geographical origins. Gas chromatography-mass spectrometry (GC-MS) analysis identified a total of 53 VOCs in the tested samples, which were categorized into 5 groups with varying compound numbers (**Figure 2A**). Monoterpenoids accounted for the largest proportion (25), followed by sesquiterpenoids (12), lipids (7), aromatic compounds (6), and other compounds (3).

Preliminary principal component analysis (PCA) was performed based on the VOC fingerprint spectra of *P. asiatica* seeds samples (**Figure 2B**). The score plot demonstrated clear separation between samples from Jiangxi and Sichuan production areas, indicating significant differences in the volatile component profiles of *P. asiatica* seeds from these two regions. To further characterize the specific differential components among origins, orthogonal partial least squares discriminant analysis (OPLS-DA) was conducted (**Figure 2C**). Cross-validation (n=200) revealed that the Y-intercept of Q² was negative, with both R²X and Q² exceeding 0.5, confirming the reliability of the model and the absence of overfitting (**Supplementary Figures S3A**). Differential metabolites were screened based on the criteria of variable importance in projection (VIP) > 1.0, P < 0.05, and fold change (FC) ≥ 2 or FC ≤ 0.5 (Huang et al., 2021), leading to the identification of 13 key differential compounds (**Supplementary Table S5**).Notably, lipid compounds in Jiangxi-sourced *P. asiatica* seeds were generally upregulated, whereas terpenoid compounds were predominantly downregulated (**Figure 2D**). Among the detected VOCs, (+)-alpha-pinene and linoleic acid were the dominant components in their respective categories (**Figure 2E**).Of particular interest, (-)-camphene and (Z)-carveol were specifically detected in Sichuan-sourced samples.

### 3.3 LC-MS analysis results

**Figure 3:**
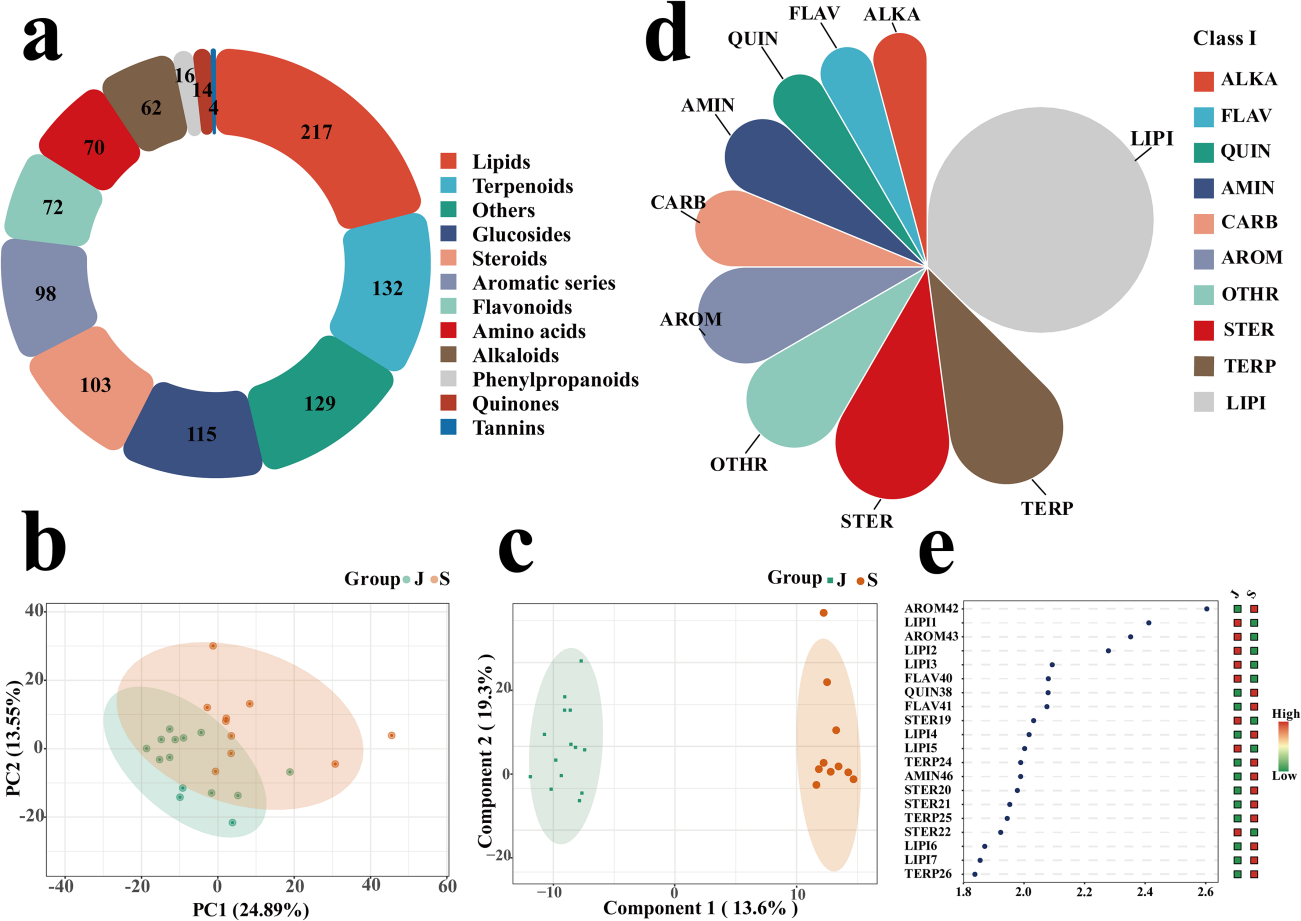
Pie charts of classification **(a)**, principal component analysis (PCA) plots **(b)**, and orthogonal projections to latent structures-discriminant analysis (OPLS-DA) plots **(c)** of non-volatile metabolites detected in Plantago asiatica L. seeds from different geographical origins. Rose pie charts **(d)** and Variable Importance in Projection (VIP) plots **(e)** of non-volatile differential metabolites detected in *Plantago asiatica L.* seeds from different geographical origins. Group “J” represents Jiangxi Province and Group “S” represents Sichuan Province.

Metabolic profiling data of *P. asiatica* seeds samples were acquired in both electrospray ionization (ESI) positive and negative ion modes. The raw data were subjected to peak extraction and normalization using MS-DIAL software, leading to the identification of 1032 metabolites that were categorized into 12 classes with distinct quantities. Lipids constituted the most abundant class (217 metabolites), followed by terpenoids (132) and other compounds (129). Glycosides (115), steroids (103), and aromatic compounds (98) formed the second tier, while flavonoids (72), amino acids and their derivatives (70), and alkaloids (62) were present in moderate amounts. The remaining classes—phenylpropanoids (16), quinones (14), and tannins (4)—were the least abundant (**Figure 3A**).

Principal component analysis (PCA) was performed based on the 1032 detected metabolites to evaluate the overall metabolic differences among samples (**Figure 3B**). In the unsupervised PCA model, samples from the two geographical origins were distinctly clustered and well separated, with the first two principal components (PC1 and PC2) explaining 24.27% and 13.41% of the total variance, respectively. After further analysis using the supervised orthogonal partial least squares discriminant analysis (OPLS-DA) model (**Figure 3C**; **Supplementary Figure S3B**), the separation effect between groups was more pronounced, with samples from Jiangxi and Sichuan forming two clear, discrete clusters. This result confirmed distinct differences in the chemical composition of *P. asiatica* seeds from different producing areas. Forty-eight key differential metabolites were screened using the criteria of variable importance in projection (VIP) > 1.0, t-test result (P < 0.05), and fold change (FC) ≥ 2 or FC ≤ 0.5 (**Supplementary Table S6**; **Figure 3D**). These differential metabolites were predominantly classified into lipids, terpenoids, and steroids—three classes of compounds closely associated with the bioactivity of *P. asiatica* seeds. Statistical analysis revealed that among these 48 metabolites, 18 were upregulated and 30 were downregulated in Jiangxi-sourced *P. asiatica* seeds compared to *P. asiatica* seeds from Sichuan. Notably, terpenoid compounds, a major class of bioactive constituents in *P. asiatica* seeds, exhibited a general downregulated trend in samples from the Jiangxi origin (**Figure 3E**).

### 3.4 Results of climatic factors analysis

**Figure 4:**
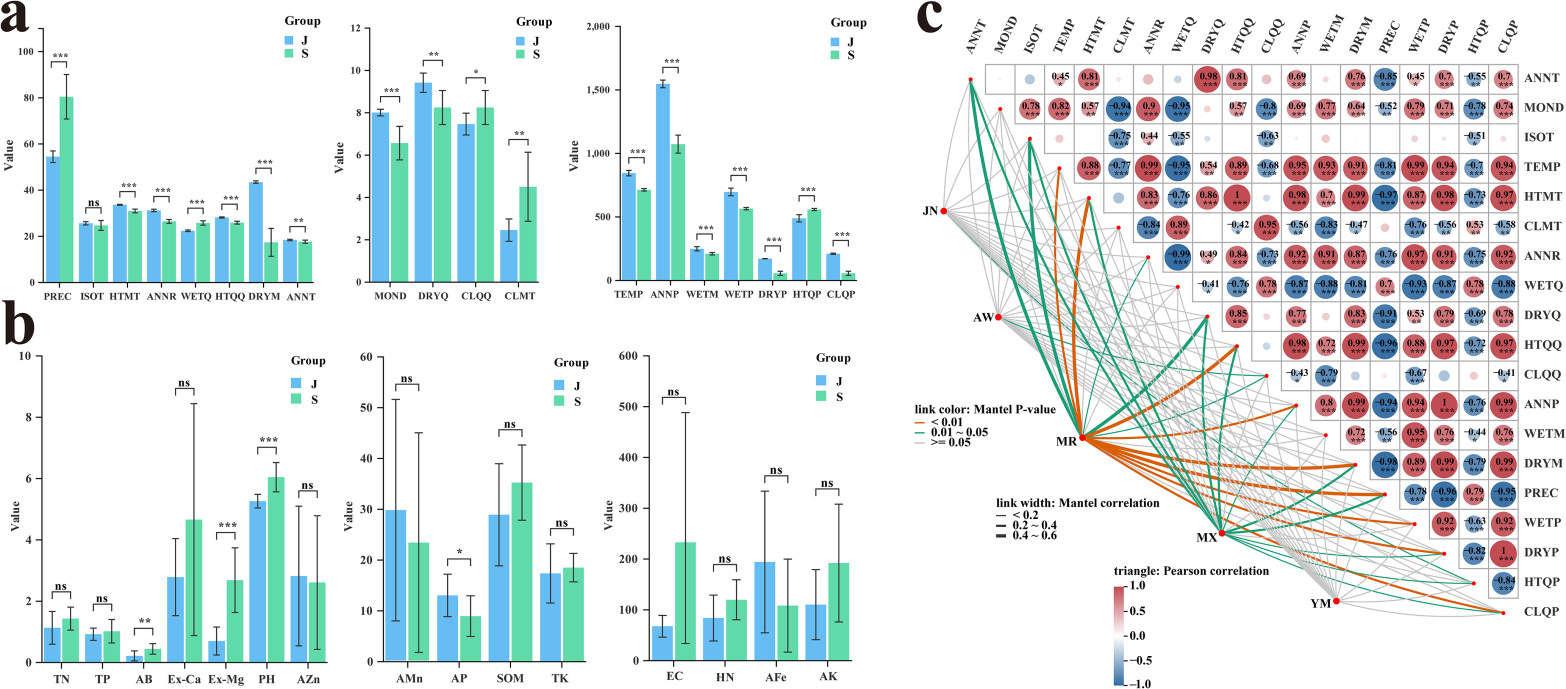
Bar chart of 19 climate factors **(A)**, bar chart of 15 soil physical and chemical indicators **(B)**, Mantel-test heat map of 19 climate factors and medicinal active components of *Plantago asiatica L.*seeds **(C)**. Group “J” represents Jiangxi Province and Group “S” represents Sichuan Province.

To clarify the potential correlation between habitat climatic conditions and the chemical composition differences of *P. asiatica* seeds, 19 climatic factors from 26 sampling sites (covering the core producing areas of Jiangxi and Sichuan) were collected and subjected to statistical analysis (**Supplementary Table S2**). The results revealed notable regional differentiation in climatic factors between the two production areas (**Figure 4A**), which may serve as a key driver of the metabolic variations of *P. asiatica* seeds. In terms of precipitation-related factors, the precipitation of the driest month, annual precipitation, precipitation of the driest quarter, and precipitation of the coldest quarter in Jiangxi were all markedly higher than those in Sichuan (all p < 0.01). This indicates that Jiangxi has more abundant overall moisture conditions, and its seasonal precipitation distribution is more conducive to maintaining a humid growth environment for *P. asiatica* seeds. In contrast, Sichuan showed two distinct climatic characteristics: the coefficient of variation of seasonal precipitation and the minimum temperature of the coldest month were notably higher than those in Jiangxi (both p < 0.05). These results suggest that Sichuan has weaker seasonal stability of precipitation and lower extreme low temperatures in winter, which may impose more fluctuating environmental constraints on the growth of *P. asiatica* seeds. Overall, the temperature in Jiangxi Province is higher, and precipitation is greater and more evenly distributed. In Sichuan, the temperature is relatively mild, but there is a significant seasonal variation in precipitation.

### 3.5 Results of soil factor analysis

To explore the potential association between soil nutrient supply and the geographical variations in chemical composition of *P. asiatica* seeds from the core production areas of Jiangxi and Sichuan, 15 soil physico-chemical indicators across 26 sampling sites were determined and subjected to statistical analysis. The results revealed notable regional differentiation in soil factors between the two areas (**Figure 4B**). The average contents of available iron (AFe), available manganese (AMn), and available phosphorus (AP) in the soil of Jiangxi production area were markedly higher than those in Sichuan production area (all p < 0.05). This suggests that the soil in Jiangxi has higher bioavailability of iron, manganese, and phosphorus, which can more effectively meet the demand of *P. asiatica* seeds for these mineral nutrients essential for plant growth and secondary metabolism. In contrast, the contents of available boron (AB), exchangeable magnesium (Ex-Mg), and the pH value in the soil of Sichuan production area were considerably higher than those in Jiangxi (all p < 0.01). These findings indicate that the soil in Sichuan has better bioavailability of boron and higher exchangeability of magnesium, forming a soil background that was significantly distinct from that of Jiangxi.

### 3.6 Correlation analysis between climatic factors and HPLC-identified key bioactive components

To clarify the climate-driven mechanisms underlying the content variations of the five core bioactive components identified by HPLC, Pearson correlation analysis combined with Mantel test was used (significance was defined as P < 0.05, and extreme significance as P < 0.01) to explore the specific response patterns of these components to 19 climatic factors (**Figure 4C**).

The results are summarized as follows: geniposidic acid was correlated solely with the precipitation of the hottest season, indicating that its content is weakly regulated by climatic factors. In contrast, acteoside had highly notable correlations with multiple climatic factors, including the maximum temperature of the hottest month, average temperature of the hottest quarter, precipitation of the driest month, coefficient of variation of seasonal precipitation, annual average temperature, temperature seasonality (isothermality), and average temperature of the driest quarter. These findings suggest that seasonal fluctuations in hydrothermal conditions are the core climatic factors regulating the accumulation of acteoside. Luteoloside was significantly correlated with annual average temperature, temperature seasonality (isothermality), maximum temperature of the hottest month, average temperature of the driest quarter, average temperature of the hottest quarter, precipitation of the driest month, and coefficient of variation of seasonal precipitation. Its accumulation is mainly regulated by annual average temperature and the combination of hydrothermal conditions during dry and wet seasons. Ferulic acid was only correlated with the average temperature of the coldest quarter, showing that its response specificity to climatic factors was relatively low. Notably, isoacteoside exhibited no correlation with any of the tested climatic factors, implying that its content may be more strongly regulated by genetic factors or non-climatic environmental cues.

### 3.7 Correlation analysis between climatic factors and metabolome

Using *P. asiatica* seeds from regions with distinct climatic conditions as research subjects, gas chromatography-mass spectrometry (GC-MS) and liquid chromatography-mass spectrometry (LC-MS) techniques were used to identify and screen differential metabolites. Pearson correlation analysis combined with Mantel test was further used to explore the specific response patterns of these differential metabolites to 19 climatic factors, with significance defined as P < 0.05 and extreme significance as P < 0.01.

#### 3.7.1 Correlation analysis between GC-MS-identified differential metabolites and climatic factors

**Figure 5:**
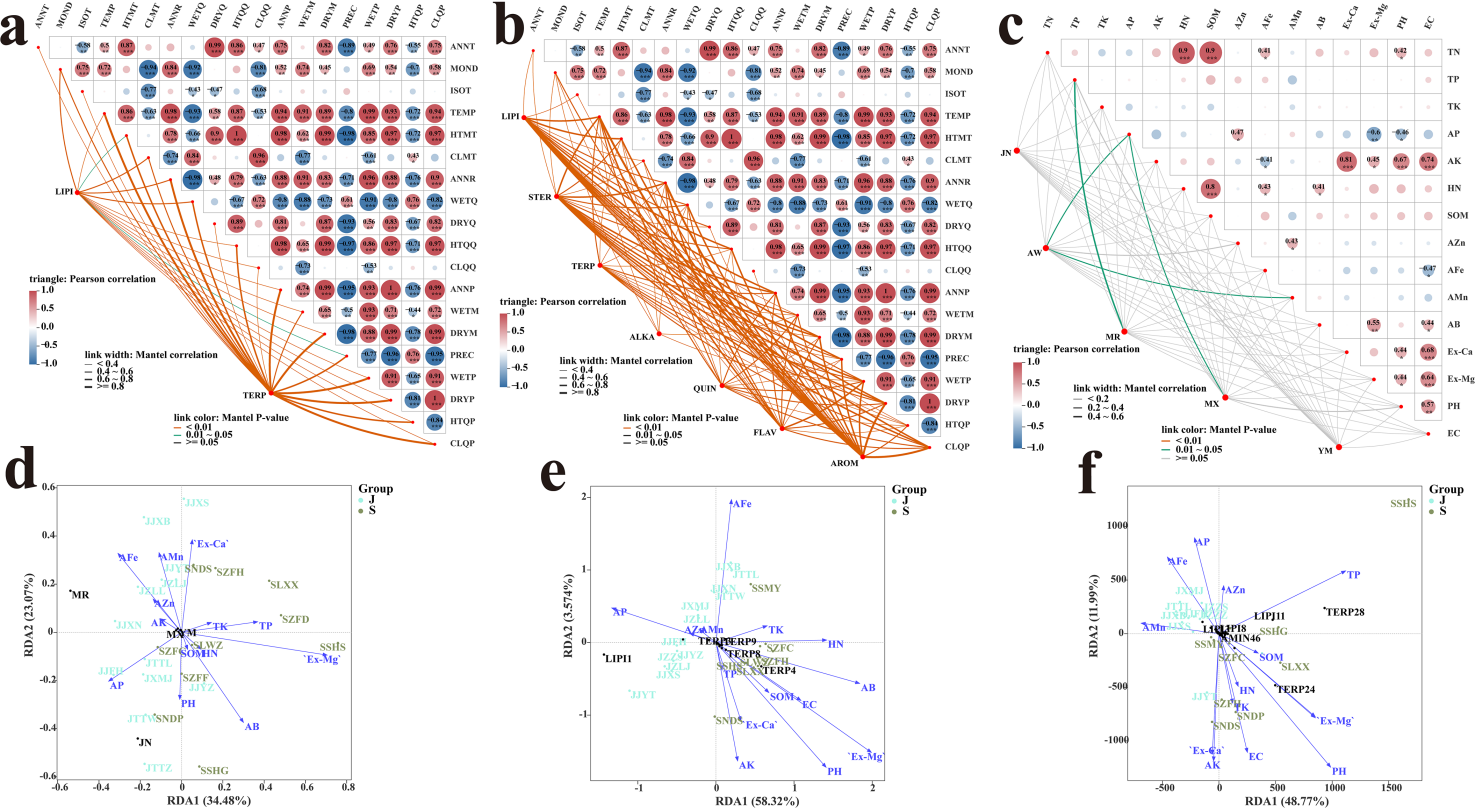
Mantel-test heat map of volatile differential metabolites and climatic factors **(A)**, Mantel-test heat map of non-volatile differential metabolites and climatic factors **(B)**, Mantel-test heat map of medicinal active ingredients and soil physical and chemical indicators **(C)** redundancy analysis **(D)**,redundancy analysis of volatile metabolites and soil physicochemical indicators **(E)**, redundancy analysis of non-volatile metabolites and soil physicochemical indicators **(F)**.

Terpenoids were extremely significantly correlated with seven climatic factors, including the standard deviation of seasonal temperature variation, annual temperature range, annual precipitation, precipitation of the driest month, precipitation of the wettest quarter, precipitation of the driest quarter, and precipitation of the coldest quarter (all p < 0.01). As the most responsive category of volatile metabolites to climatic hydrothermal conditions, the accumulation of terpenoids was primarily regulated by temperature fluctuation amplitude and precipitation during cold seasons. Lipids were extremely significantly correlated with four climatic factors: mean diurnal temperature range, minimum temperature of the coldest month, annual temperature range, and mean temperature of the wettest quarter (all p < 0.01). The response pattern of lipids to climatic factors was similar to that of terpenoids to some extent, with both being dependent on warm-season temperature conditions and annual precipitation distribution characteristics (**Figure 5A**).

#### 3.7.2 Correlation analysis between LC-MS-identified differential metabolites and climatic factors

Terpenoids were extremely significantly correlated with 13 climatic factors, namely the standard deviation of seasonal temperature variation, maximum temperature of the hottest month, annual temperature range, mean temperature of the wettest quarter, mean temperature of the hottest quarter, annual precipitation, precipitation of the wettest month, precipitation of the driest month, coefficient of variation of seasonal precipitation, precipitation of the wettest quarter, precipitation of the driest quarter, precipitation of the hottest quarter, and precipitation of the coldest quarter (all p < 0.01).

Lipids were extremely significantly correlated with eight climatic factors: standard deviation of seasonal temperature variation, maximum temperature of the hottest month, mean temperature of the hottest quarter, annual precipitation, precipitation of the driest month, coefficient of variation of seasonal precipitation, precipitation of the driest quarter, and precipitation of the coldest quarter (all p < 0.01).Steroids were extremely significantly correlated with seven climatic factors, including standard deviation of seasonal temperature variation, annual temperature range, annual precipitation, precipitation of the driest month, precipitation of the wettest quarter, precipitation of the driest quarter, and precipitation of the coldest quarter (all p < 0.01).Alkaloids were extremely significantly correlated with six climatic factors: mean diurnal temperature range, standard deviation of seasonal temperature variation, annual temperature range, mean temperature of the wettest quarter, precipitation of the wettest month, and precipitation of the wettest quarter (all p < 0.01).

Quinones were extremely significantly correlated with seven climatic factors: standard deviation of seasonal temperature variation, annual temperature range, annual precipitation, precipitation of the wettest month, precipitation of the wettest quarter, precipitation of the driest quarter, and precipitation of the coldest quarter (all p < 0.01).Flavonoids were extremely significantly correlated with three climatic factors: standard deviation of seasonal temperature variation, annual temperature range, and precipitation of the wettest quarter (all p < 0.01).Aromatic compounds were extremely significantly correlated with four climatic factors: standard deviation of seasonal temperature variation, annual temperature range, precipitation of the driest quarter, and precipitation of the coldest quarter (all p < 0.01).Metabolites in the categories of sugars, amino acids and their derivatives, and others were excluded from subsequent correlation analysis due to their relatively weak associations with climatic factors (**Figure 5B**).

### 3.8 Correlation analysis between soil factors and HPLC-identified key bioactive components

To elucidate the soil-driven mechanisms underlying the content variations of the five core bioactive components detected by HPLC, Pearson correlation analysis, Mantel test, and redundancy analysis (RDA) were combined to investigate the association patterns between these five core bioactive components and 15 soil physico-chemical properties. Statistical significance was defined as P < 0.05, and extreme significance as P < 0.01.

#### 3.8.1 Differences in response characteristics of HPLC-identified key bioactive components to soil factors

Pearson correlation analysis results (**Figure 5C**) revealed that different bioactive components exhibited significant specificity in their responses to soil factors, reflecting the selective regulatory effects of soil properties on the accumulation of *P. asiatica* seeds’ active constituents. Acteoside was significantly and positively correlated with available phosphorus (AP) and available manganese (AMn) (all p < 0.05). This finding indicates that enhancing the bioavailability of phosphorus and manganese in the soil could promote the accumulation of acteoside in *P. asiatica* seeds. Luteoloside was significantly and positively correlated with available phosphorus (AP) (p < 0.05), exhibiting a more specific response to soil phosphorus availability compared with other components. Ferulic acid was extremely significantly and positively correlated with total phosphorus (TP) (p < 0.01), and it was the only bioactive component regulated by total phosphorus content among the five core components, highlighting its unique response pattern to soil phosphorus pools. Geniposidic acid and isoacteoside showed no significant correlation with any of the 15 soil physico-chemical indicators (all p > 0.05), suggesting that the contents of these two components are weakly regulated by soil factors and may be more dependent on other environmental cues or genetic traits.

#### 3.8.2 Redundancy analysis between soil factors and HPLC-identified key bioactive components

The RDA results (**Figure 5D**) showed that the first two ordination axes collectively explained 57.55% of the total variation in the five key HPLC-identified bioactive components, indicating a good fitting degree of the RDA model. Among the explanatory variables, exchangeable magnesium (Ex-Mg), available boron (AB), available phosphorus (AP), and available iron (AFe) were identified as the core soil factors that regulate the bioactive components. Notably, exchangeable magnesium (Ex-Mg) was significantly correlated with the overall variation of bioactive components (p = 0.033).

### 3.9 Correlation analysis between soil factors and metabolome

#### 3.9.1 Redundancy analysis between GC-MS-identified differential metabolites and soil factors

The RDA results (**Figure 5E**) showed that the first two ordination axes collectively explained 61.89% of the total variation in the volatile metabolome of *P. asiatica* seeds, indicating a good fitting degree of the RDA model. Three soil factors were identified as core driving factors: available iron (AFe, p = 0.008), available boron (AB, p = 0.002), and exchangeable magnesium (Ex-Mg, p = 0.001), all of which were extremely significantly correlated with the volatile metabolome of *P. asiatica* seeds (all p < 0.01). Specifically, lipid metabolites LIPI1 and LIPI2 were positively correlated with available phosphorus (AP) and available manganese (AMn). Additionally, LIPI2 was positively correlated with AFe. This correlation pattern was consistent with the soil characteristics of Jiangxi Province, suggesting that the relatively high contents of AP, AMn, and AFe in Jiangxi’s soil may contribute to the upregulation of lipid metabolites in local *P. asiatica* seeds. In contrast, most terpenoid metabolites were positively correlated with AB, Ex-Mg, and soil pH, which aligned with the soil properties of Sichuan Province.

#### 3.9.2 LC-MS differential metabolites and RDA analysis of soil factors

The results (**Figure 5F**) showed that the first two axes of the RDA collectively accounted for 60.76% of the total variance in the LC-MS-detected metabolome, indicating a good fit of the RDA model. The core driving factors were identified as total phosphorus (TP, p = 0.038), available potassium (AK, p = 0.019), exchangeable calcium (Ex-Ca, p = 0.035), electrical conductivity (EC, p = 0.036), and pH (p = 0.001). Among these factors, TP, AK, Ex-Ca, and EC showed a notable correlation with the metabolome (p < 0.05), while pH exhibited a highly notable correlation (p < 0.01). Notably, a subset of lipid metabolites showed a positive correlation with available phosphorus (AP), available manganese (AMn), and available iron (AFe). This observation is consistent with the soil properties of Jiangxi Province, suggesting that the high levels of AP, AMn, and AFe in Jiangxi’s soil may contribute to the upregulation of lipids in *P. asiatica* seeds from this region. Most terpenoid metabolites showed a positive correlation with available boron (AB), exchangeable magnesium (Ex-Mg), and pH, which aligns with the soil characteristics of Sichuan Province.

#### 3.9.3 GC-MS differential metabolites and mantel-test correlation analysis of soil factors

**Figure 6:**
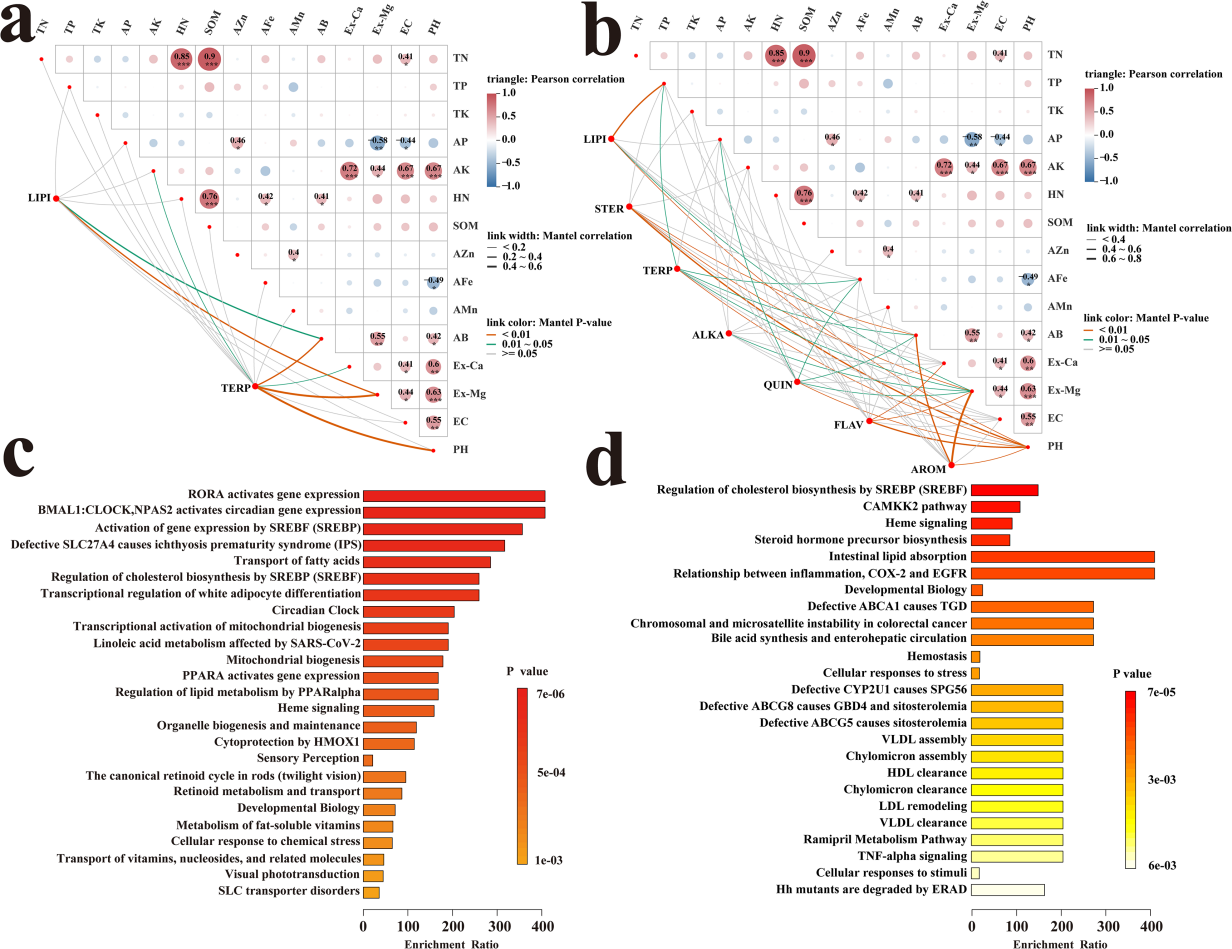
Mantel-tset heat maps of volatile metabolites and soil physical and chemical indicators **(A)**, Mantel-tset heat maps of non-volatile metabolites and soil physical and chemical indicators **(B)**, KEGG enrichment analysis diagrams of volatile metabolites **(C)**, and KEGG enrichment analysis diagrams of non-volatile metabolites **(D)**.

The results (**Figure 6A**) indicated that terpenoids (TERP) in the temperament-related metabolome were a category with strong responsiveness. Specifically, TERP were highly notably correlated with available boron (AB), exchangeable magnesium (Ex-Mg), and pH (p < 0.01), and significantly correlated with available potassium (AK) (p < 0.05). Additionally, lipids (LIPI) were highly significantly correlated with Ex-Mg (p < 0.01) and significantly correlated with AB (p < 0.05).

#### 3.9.4 LC-MS differential metabolites and mantel-test correlation analysis of soil factors

The results (**Figure 6B**) revealed distinct correlation patterns between different metabolome categories and soil factors. Specifically, terpenoids (TERP) were highly significantly correlated with pH (p < 0.01), and significantly correlated with total phosphorus (TP), available iron (AFe), available boron (AB), and exchangeable magnesium (Ex-Mg) (p < 0.05). Lipids (LIPI) were highly significantly correlated with TP and pH (p < 0.01), and notably correlated with Ex-Mg (p < 0.05). Steroids (STER) showed highly significant correlations with AB, Ex-Mg, and pH (p < 0.01), while alkaloids (ALKA) were significantly correlated with Ex-Mg (p < 0.05). Quinones (QUIN) were highly significantly correlated with pH (p < 0.01), and significantly correlated with AB, Ex-Mg, AFe, and available phosphorus (AP) (p < 0.05). Notably, flavonoids (FLAV) were highly significantly correlated with exchangeable calcium (Ex-Ca), Ex-Mg, and pH (p < 0.01), and aromatic compounds (AROM) also exhibited highly significant correlations with AB, Ex-Mg, and pH (p < 0.01).

### 3.10 KEGG pathway enrichment analysis of differential metabolites

To gain insights into the molecular mechanisms underlying the metabolomic differences of *P. asiatica* seeds between Jiangxi and Sichuan provinces, KEGG pathway enrichment analysis was performed on the differential metabolites screened by gas chromatography-mass spectrometry (GC-MS) and liquid chromatography-mass spectrometry (LC-MS), respectively.

#### 3.10.1 KEGG pathway enrichment analysis of GC-MS-identified differential metabolites

The results (**Figure 6C**) showed that the differential metabolites identified by GC-MS were significantly enriched in four core pathways, namely Linoleic acid metabolism (ko00591), Fatty acid transport (ko04972), PPARα-regulated lipid metabolism (ko03320), and Retinoid metabolism and transport (ko04996). Among these pathways, linoleic acid—a differentially accumulated metabolite—was significantly upregulated in Jiangxi-sourced *P. asiatica* seeds, while its downstream product arachidonic acid was present at a lower content compared to Sichuan-sourced seeds. The PPARα-regulated lipid metabolism pathway was differentially enriched between the two regions. Additionally, the differential metabolites enriched in the retinoid metabolism and transport pathway were primarily terpene derivatives, which were consistent with the high proportion of terpenoids among GC-MS-identified differential metabolites.

#### 3.10.2 KEGG pathway enrichment analysis of LC-MS-identified differential metabolites

The results (**Figure 6D**) showed that the differential metabolites identified by LC-MS were significantly enriched in four core pathways, namely Steroid hormone precursor biosynthesis (ko00140), Intestinal lipid absorption (ko04977), SREBP-mediated regulation of cholesterol biosynthesis (ko04979), and the COX-2-EGFR-associated inflammatory pathway (ko04064). The enrichment of the steroid hormone precursor biosynthesis pathway was consistent with the high proportion of steroids among LC-MS-identified differential metabolites. Notably, the COX-2-EGFR-associated inflammatory pathway was differentially enriched between the two regions, which was in line with the previous HPLC analysis result that acteoside content was higher in Jiangxi-sourced *P. asiatica* seeds. Additionally, the SREBP-mediated regulation of cholesterol biosynthesis pathway and the intestinal lipid absorption pathway were also differentially enriched, indicating systematic differentiation in the lipid metabolism network of *P. asiatica* seeds from Jiangxi and Sichuan.

## 4 Discussion

Metabolomic detection results revealed that *P. asiatica* seeds from Jiangxi and Sichuan provinces exhibited significant differences in core active components, volatile metabolites, and non-volatile metabolites. High-performance liquid chromatography (HPLC) analysis confirmed that the contents of geniposidic acid, acteoside, and luteoloside in *P. asiatica* seeds from Jiangxi were generally higher than those from Sichuan. All three components are key contributors to the anti-inflammatory and antioxidant activities of *P. asiatica* seeds, and their superior content directly supports the traditional recognition that “Jiangxi-sourced *P. asiatica* seeds possess superior medicinal quality”. This finding is consistent with the “enrichment rule of core active components in genuine medicinal materials” documented in existing studies (Li et al., 2020; Sun et al., 2025). Moreover, combined GC-MS and LC-MS analysis further revealed that the metabolomic differences between the two regional samples were primarily concentrated in lipids and terpenoids: lipids in Jiangxi-sourced seeds were generally upregulated, whereas Sichuan-sourced seeds exhibited significant enrichment of terpenoids and contained unique volatile organic compounds (VOCs) such as (-)-camphene and (Z)-carveol. These unique VOCs could serve as characteristic markers for the geographical traceability of *P. asiatica* seeds.

Climatic and ecological factors, including temperature and precipitation, regulate the accumulation of secondary metabolites by affecting plant enzyme activities and metabolic pathway fluxes, and are key drivers of medicinal quality formation in medicinal plants (Daneshmandi et al., 2025; Sun et al., 2023; Wei et al., 2024). The climatic characteristics of high temperature, abundant precipitation, and uniform precipitation distribution in Jiangxi were significantly correlated with the upregulation of lipid metabolites in *P. asiatica* seeds (Couvillion et al., 2023), whereas the climatic conditions in Sichuan—characterized by mild temperatures and significant seasonal precipitation variations—were more favorable for the accumulation of terpenoid compounds. This result conforms to the adaptive principle of plant secondary metabolism: abundant and stable hydrothermal resources can promote the synthesis of energy metabolism-related substances (e.g., lipids) (Couvillion et al., 2023), whereas seasonal environmental fluctuations can induce the accumulation of stress-resistant secondary metabolites (e.g., terpenoids) (Qaderi et al., 2023b). Acteoside, a core active component of *P. asiatica* seeds, exhibits a strong correlation with multiple climatic factors, including the maximum temperature in the hottest month and precipitation variations between dry and wet seasons. This suggests that its accumulation is highly sensitive to seasonal fluctuations in hydrothermal conditions, which thus also explains why the stable hydrothermal environment in Jiangxi is more conducive to the enrichment of this active component.

As the growth substrate for plants, soil physical and chemical properties indirectly regulate the synthesis of metabolites by influencing the absorption of mineral nutrients and the root microenvironment. The regulatory mechanisms of soil differences between the producing areas of Jiangxi and Sichuan on metabolite accumulation are distinct and specific. In Jiangxi, soil characteristics of high available phosphorus (AP), available iron (AFe), and available manganese (AMn) were significantly and positively correlated with the enrichment of lipid metabolites (especially linoleic acid) and anti-inflammatory active components (acteoside) (P < 0.05). Specifically, available phosphorus provides the energy and material basis for the accumulation of secondary metabolic precursors (Yang et al., 2024); available iron participates in hydroxylation and oxidation reactions during phenylethanoid glycoside synthesis (Baruah et al., 2024); and available manganese, as a cofactor of flavonoid synthase, enhances enzymatic reaction efficiency. Collectively, these factors not only may potentially regulate the linoleic acid metabolic pathway (Lakk-Bogáth et al., 2021) but also potentially inhibit arachidonic acid biosynthesis—likely via competing for common C18 fatty acid precursors (e.g., linoleic acid itself) or suppressing the activity of Δ6-desaturase, an evolutionarily conserved rate-limiting enzyme that catalyzes the conversion of linoleic acid to γ-linolenic acid (the first step in arachidonic acid synthesis) (Hanna and Hafez, 2018), thereby leading to lower arachidonic acid content in Jiangxi samples. In contrast, the high available boron (AB), exchangeable magnesium (Ex-Mg), and slightly alkaline soil in Sichuan may be involved in modulating the retinoid metabolism and transport pathway, thereby promoting terpenoid accumulation and the production of characteristic components such as (-)-camphene. Among these factors, available boron (AB) stabilizes cell membrane structure and participates in sugar transport, providing structural support and carbon source guarantee for terpenoid synthesis; additionally, its regulation of isoprene precursor transport constitutes a key link in promoting terpenoid accumulation (Blevins and Lukaszewski, 1998). The slightly alkaline pH environment not only enhances the availability of nutrients (e.g., boron and magnesium) by altering the surface charge properties of soil colloids but also regulates root proton pump activity and thereby affects the conformation of metabolic enzymes, thereby directionally activating the retinoid metabolism pathway (Cazzonelli and Pogson, 2010). As a core component of chlorophyll, exchangeable magnesium (Ex-Mg) ensures photosynthetic efficiency to supply sufficient metabolic substrates; furthermore, as an activator of various metabolic enzymes (especially key enzymes for terpenoid synthesis), it plays a central role in the regulation of metabolic pathways (Chen et al., 2018; Gong et al., 2020).

Beyond quantitative differences, qualitative and pathway-specific variations in lipids and terpenoids further shape the medicinal efficacy of *P. asiatica* seeds by regulating distinct biological processes. For lipids enriched in Jiangxi-sourced seeds, linoleic acid (an essential unsaturated fatty acid) synergizes with anti-inflammatory components (e.g., acteoside) rather than accumulating as a metabolic byproduct: it may modulate the PPARα-regulated lipid metabolism pathway (Goto et al., 2010) to modulate lipid homeostasis and inhibit proinflammatory mediator release (e.g., TNF-α, IL-6), reinforcing acteoside’s anti-inflammatory activity via the COX-2-EGFR pathway (ko04064). Additionally, enrichment of the intestinal lipid absorption pathway (ko04977) is inferred to improve the bioavailability of lipid-soluble active components, ensuring efficient delivery of anti-inflammatory metabolites and explaining the superior efficacy of Jiangxi-sourced seeds in alleviating inflammatory disorders (e.g., urinary and digestive inflammation). For terpenoids predominantly accumulated in Sichuan-sourced seeds, their medicinal value extends to functional diversification: unique (-)-camphene and (Z)-carveol not only enable geographical traceability but also exert complementary pharmacological effects, with (-)-camphene inhibiting bacterial proliferation by disrupting microbial membrane integrity (Quintans-Júnior et al., 2013) and (Z)-carveol exerting sedative and analgesic activities via modulating neurotransmitter release (Kpoviessi et al., 2014). Notably, the distinct modulation of terpenoid and lipid pathways leads to functional specialization: Jiangxi-sourced seeds are optimized for anti-inflammatory therapy, while Sichuan-sourced seeds exhibit multifaceted efficacy in anti-infection, analgesia, and sedation.

**Figure 7:**
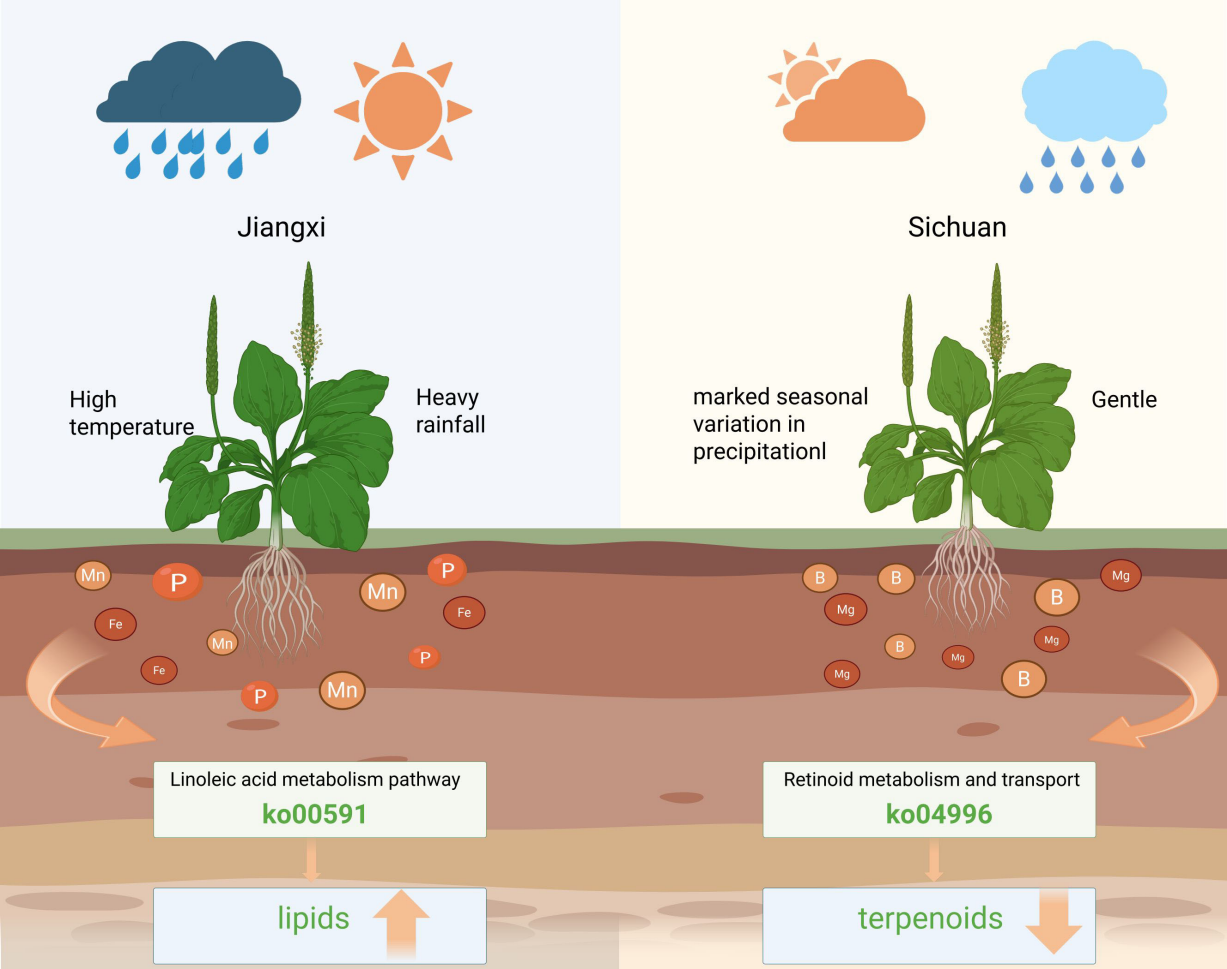
Prediction graph of the mechanism by which environmental factors affect the quality of *Plantago asiatica L.*seeds.

Integrated analysis revealed that the metabolomic differences in *P. asiatica* seeds result from the synergistic effects of climate and soil factors. Specifically, in the Jiangxi producing area, the combination of a climate with high temperature and abundant rainfall and soils rich in available phosphorus (AP), available iron (AFe), and available manganese (AMn) may facilitate the enrichment of lipids and anti-inflammatory active components by activating pathways such as linoleic acid metabolism. In contrast, the Sichuan producing area features a mild climate with seasonal fluctuations coupled with alkaline soils high in available boron (AB) and exchangeable magnesium (Ex-Mg); this combination may enable the specific accumulation of terpenoids by regulating the retinoid metabolism and transport pathways (**Figure 7**). Notably, the regulatory network of “environmental factors → metabolic pathways → differential metabolites” is consistent with the well-documented mechanism of “environmental conditions modulate the quality of Chinese medicinal materials by regulating key metabolic pathways” (Zhang et al., 2025). Furthermore, the differential enrichment of the COX-2/EGFR-associated inflammation-related pathway further unveils the molecular essence that underlies the differences in anti-inflammatory activity of *P. asiatica* seeds between the two regions.

## 5 Conclusions

This study predicted the core metabolomic differences and regulatory mechanisms of *P. asiatica* seeds from Jiangxi and Sichuan producing areas by integrating multiple metabolomic techniques and multi-factor association validation. Specifically, *P. asiatica* seeds from Jiangxi, under the combined influence of a hot and rainy climate and soils rich in available phosphorus (AP), available iron (AFe), and available manganese (AMn), may accumulate lipids and anti-inflammatory active components (e.g., geniposidic acid, acteoside) via pathways including linoleic acid metabolism, endowing them with superior medicinal quality. In contrast, *P. asiatica* seeds from Sichuan, under the synergistic influence of a mild climate with significant seasonal precipitation fluctuations and soils characterized by high available boron (AB), exchangeable magnesium (Ex-Mg), and slightly alkaline properties, may achieve significant enrichment of terpenoids and contain unique volatile components (e.g., (-)-camphene and (Z)-carveol) by regulating the retinoid metabolism and transport pathways.

It should be noted that the current conclusions regarding the “activation” of the linoleic acid pathway and the “regulation/activation” of the retinoid metabolism and transport pathway lack direct evidence such as enzyme activity or gene expression data, and we are conducting relevant verification work. For future research, we propose the following testable hypotheses to further validate the regulatory mechanisms: (1) Hypothesis 1: The high temperature, abundant precipitation, and soil high in AP/AFe/AMn in Jiangxi activate the linoleic acid pathway by specifically upregulating the expression of key genes (e.g., FAD2, LOX2) and enhancing the activity of rate-limiting enzymes (e.g., fatty acid desaturase, lipoxygenase) in this pathway; (2) Hypothesis 2: The mild climate with seasonal precipitation fluctuations, soil high in AB/Ex-Mg, and slight alkalinity in Sichuan promote terpenoid accumulation by upregulating the expression of core genes (e.g., CRTISO, RBP2, IspD) and the activity of key enzymes (e.g., carotenoid isomerase, retinol-binding protein) in the retinoid metabolism and transport pathway; (3) Hypothesis 3: Silencing or overexpressing the above key genes in *P. asiatica* seeds will significantly alter the accumulation of linoleic acid/terpenoid metabolites and related active components (e.g., acteoside, (-)-camphene), and this alteration can be reversed by adjusting the corresponding environmental factors (e.g., supplementing AP/AFe/AMn for linoleic acid pathway, adjusting soil pH and AB/Ex-Mg content for retinoid pathway).

Collectively, the dual climate-soil factors synergistically shape the metabolomic differences and medicinal quality variation of *P. asiatica* seeds from the two regions by regulating distinct metabolic pathways. These findings lay a scientific foundation for the screening of high-quality *P. asiatica* seed producing areas, the regulation of planting environments, and standardized production, and thereby provide a demonstration model for the systematic analysis of quality formation mechanisms of genuine Chinese medicinal materials.

## Data Availability

The original contributions presented in the study are included in the article/**Supplementary Material**. Further inquiries can be directed to the corresponding authors.
