# Supplementary material for: Environmental influences on the accumulation of medicinal active components and metabolites of Plantago asiatica L. seeds from different cultivation sites
Source: Front Plant Sci. 2026 Feb 24;17:1775798. doi: 10.3389/fpls.2026.1775798 (PMC12971945; doi:10.3389/fpls.2026.1775798)
Supplement: Supplementary file 1 [file DataSheet1.docx]

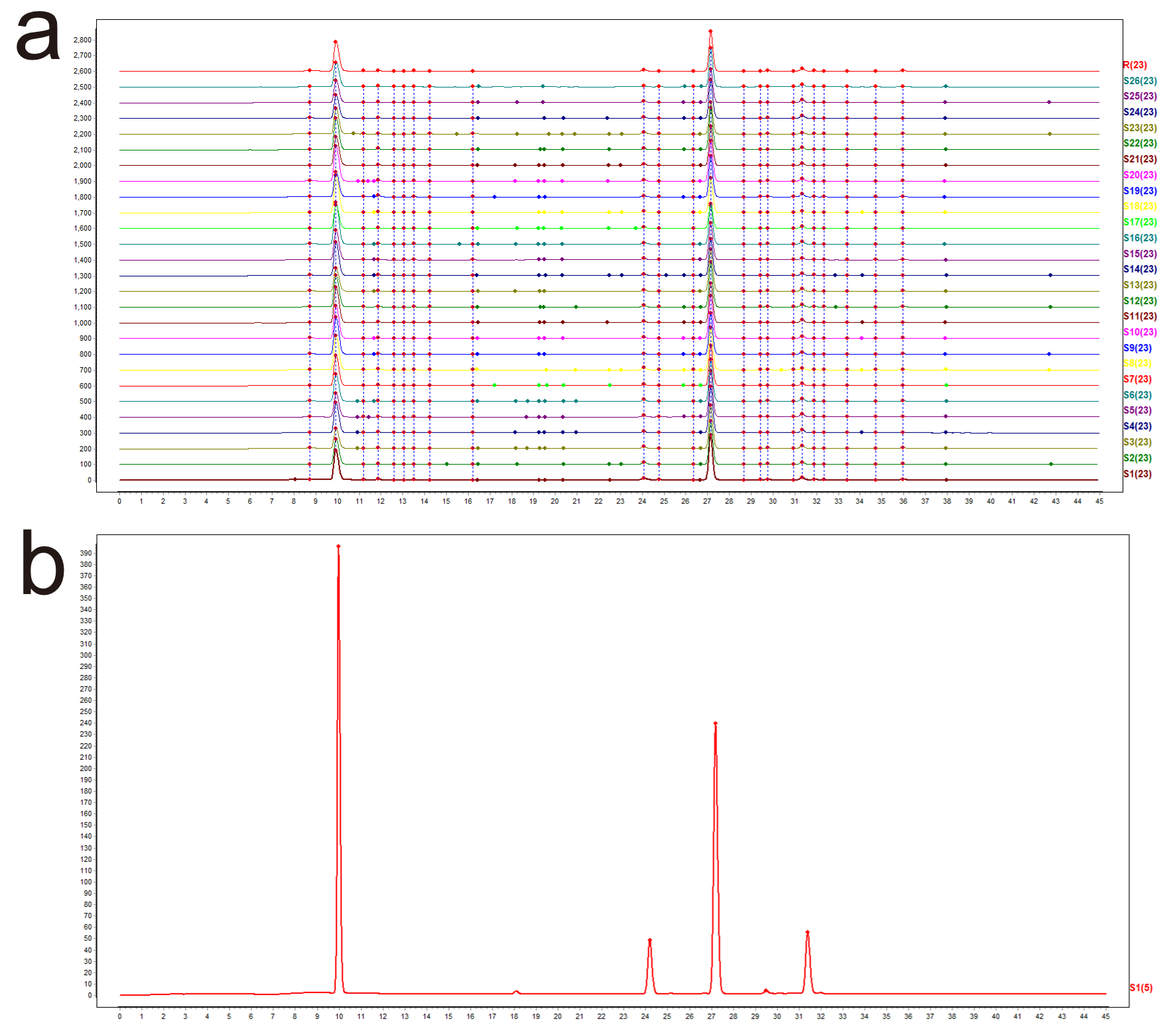


**Fig. S1** (a) HPLC chart of 23 common peaks of *Plantago asiatica L.*seeds; (b) comparison diagrams of five standard substances.


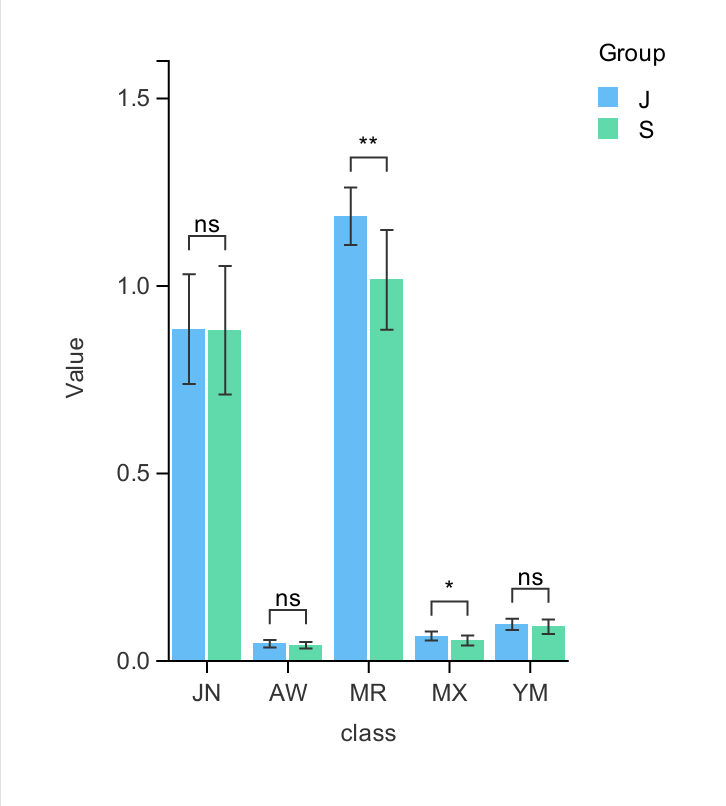


**Fig. S2** Different active ingredients in HPLC detection (JN: geniposidic acid, AW: ferulic acid, MR: acteoside, MX: luteoloside, YM: isoacteoside) and samples from two provinces (J: Jiangxi Province, S: A bar chart of numerical comparison for Sichuan Province, where "ns" indicates no significant statistical difference between groups.


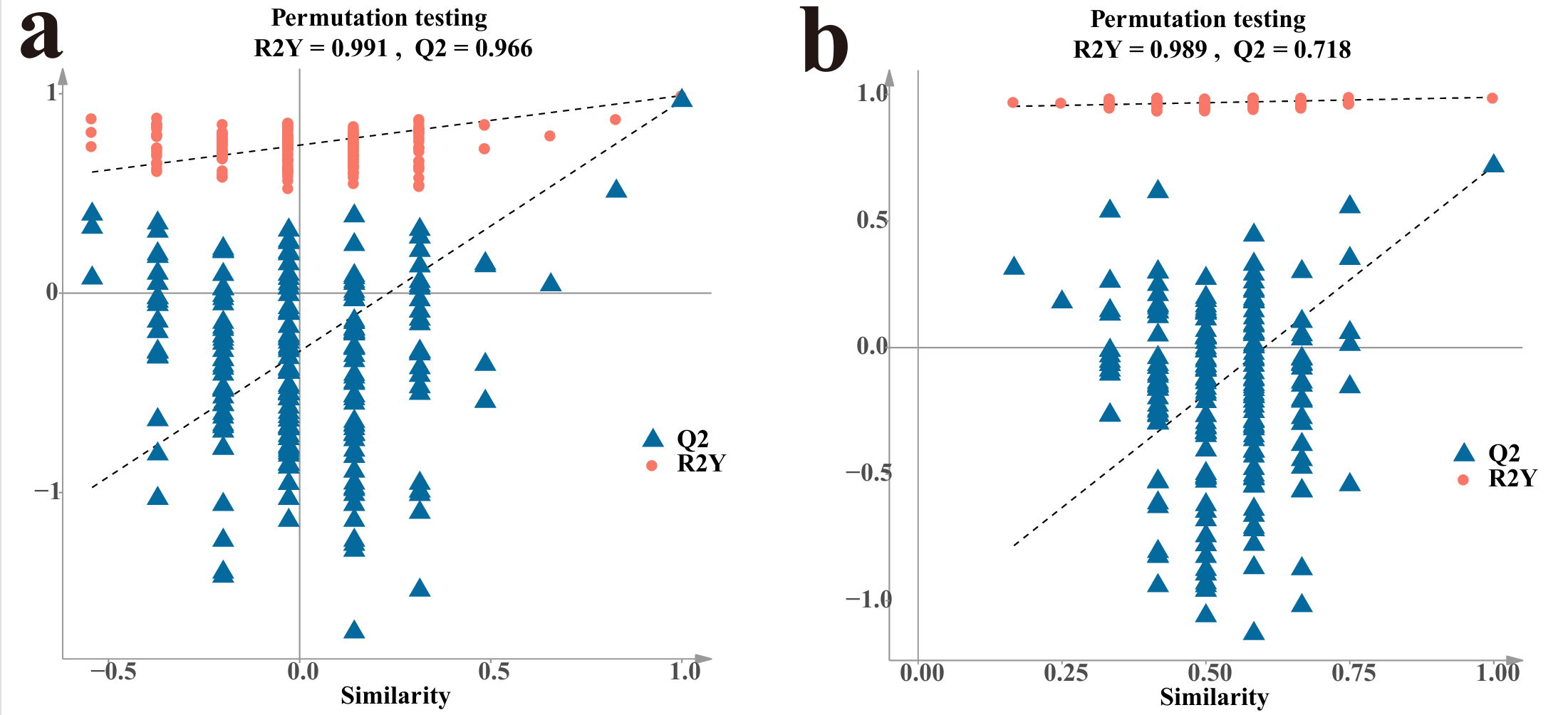


**Fig. S3** (a) Verification diagram of the OPLS-DA model of GC-MS; (b) Verification diagram of the OPLS-DA model of LC-MS.
